# Fatal primary amoebic meningoencephalitis in a Norwegian tourist returning from Thailand

**DOI:** 10.1099/jmmcr.0.005042

**Published:** 2016-06-25

**Authors:** Tore Taksdal Stubhaug, Olaug Marie Reiakvam, Christen Rune Stensvold, Nils Olav Hermansen, Mona Holberg-Petersen, Ellen-Ann Antal, Knut Gaustad, Ingrid Schage Førde, Bernt Heger

**Affiliations:** ^1^​ Department of Microbiology, Oslo University Hospital – Ullevaal, Oslo, Norway; ^2^​ Department of Infectious Diseases, Oslo University Hospital – Ullevaal, Oslo, Norway; ^3^​ Department of Microbiology and Infection Control, Statens Serum Institut, Copenhagen, Denmark; ^4^​ Department of Pathology, Oslo University Hospital – Rikshospitalet, Oslo, Norway; ^5^​ Department of Anaesthesiology, Oslo University Hospital – Ullevaal, Oslo, Norway

**Keywords:** primary amoebic meningoencephalitis, *Naegleria fowleri*, free-living amoebae, meningitis, meningoencephalitis, travel medicine, nasal irrigation

## Abstract

**Introduction:**

Primary amoebic meningoencephalitis (PAM) is a rare disease caused by the free-living amoeba *Naegleria fowleri*. Infection occurs by insufflation of water containing amoebae into the nasal cavity, and is usually associated with bathing in freshwater. Nasal irrigation is a more rarely reported route of infection.

**Case presentation:**

A fatal case of PAM in a previously healthy Norwegian woman, acquired during a holiday trip to Thailand, is described. Clinical findings were consistent with rapidly progressing meningoencephalitis. The cause of infection was discovered by chance, owing to the unexpected detection of *N. fowleri* DNA by a PCR assay targeting fungi. A conclusive diagnosis was established based on sequencing of *N. fowleri* DNA from brain biopsies, supported by histopathological findings. Nasal irrigation using contaminated tap water is suspected as the source of infection.

**Conclusion:**

The clinical presentation of PAM is very similar to severe bacterial meningitis. This case is a reminder that when standard investigations fail to identify a cause of infection in severe meningoencephalitis, it is of crucial importance to continue a broad search for a conclusive diagnosis. PAM should be considered as a diagnosis in patients with symptoms of severe meningoencephalitis returning from endemic areas.

## Introduction

Primary amoebic meningoencephalitis (PAM) is a rare disease caused by the free-living amoeba *Naegleria fowleri*. Infection occurs when amoebae penetrate the CNS via the olfactory nerve, and is traditionally associated with insufflation of freshwater into the nasal cavity during bathing (Visvesvara *et al.,* 2007). Another route of infection, only recently brought to attention, is nasal irrigation using freshwater, including tap water (Shakoor *et al.,* 2011; Siddiqui & Khan, 2014; Yoder *et al.,* 2012). The geographical range of *N. fowleri* has been expanding over the past few years (Kemble *et al.,* 2012). This report presents a case of a previously healthy woman who died of PAM contracted in Thailand, presumably through nasal irrigation.

## Case report

A 71-year-old Norwegian woman was admitted to a local hospital in Oslo only hours after returning from a four-week holiday to Thailand. She presented with rapid onset of nausea and vomiting associated with fever and exhaustion, and had also been complaining of fatigue for two days. She was febrile (39.5 °C), and had a respiratory rate of 26 min^−1^. The leukocyte count was 12×10^9^cells ml^−1^ and the C-reactive protein (CRP) level was 9 mg l^−1^. Intravenous antibiotic therapy with ampicillin and gentamicin was commenced on suspicion of urosepsis, but the patient’s condition rapidly deteriorated the same evening, with increasing confusion and speech disturbances; nuchal rigidity was noted. Bacterial meningitis now appeared likely. The patient was administered 4 g of ceftriaxone and transferred to Oslo University Hospital (OUS).

Upon arrival at OUS, the patient had obvious nuchal rigidity and incoherent speech, and was motorically agitated and confused. The Glasgow Coma Scale (GCS) score was assessed as 12, but downgraded to 9 during the initial evaluation, at which point she was intubated.

A cerebral computed tomography (CT) scan with angiography showed no acute ischaemic lesions, haemorrhage, arterial occlusion, stenosis, or aneurism. Lumbar puncture yielded cloudy cerebrospinal fluid (CSF) under increased pressure, with 0.1 mmol l^−1^ glucose (serum glucose 11.1 mmol l^−1^), a protein level of 6.9 g l^−1^, and a leukocyte count of 2115 mm^−3^ with 87.3 % neutrophils. No microbes were found on an acridine orange stain of CSF. A nigrosin stain did not show any cryptococci, although the examiner noted the presence of unidentified elements. Treatment was changed to meropenem and vancomycin since the infection was presumably acquired in a region endemic for highly resistant bacteria.

The patient’s condition deteriorated over the following twelve hours in spite of early initiation of antibiotic therapy. CRP increased to 269 mg l^−1^. A repeat cerebral CT scan showed general oedema, hydrocephalus and uncal herniation. External ventricular drainage with monitoring of intracerebral pressure (ICP) was established; ICP was measured to 80–90 mmHg. The patient remained unresponsive, with fixed dilated pupils. After observing that ICP remained consistently equal to mean arterial pressure, and examination indicated cessation of all cerebral functioning with no hope of improvement, active treatment was withdrawn three days after admission, and the patient was pronounced dead shortly thereafter. An autopsy was requested.

## Investigations

Bacterial and fungal cultures of CSF were negative, as were polymerase chain reaction (PCR) assays for possible bacterial or viral causes. Due to the absence of an identified cause and lack of response to antibiotics, cryptococcal meningitis was suspected. A cryptococcal latex antibody test was performed on CSF with an equivocal result, and pan-fungal PCR assays using primers ITS3 and ITS4 targeting fungal internal transcribed spacer 2 (ITS2) rDNA (White *et al.,* 1990, p. 317), as well as primers NL−1 and NL−4 targeting the D1/D2 region of fungal 28S rDNA (Kurtzman & Robnett, 1997), were performed. The D1/D2 assay was positive, yielding a 572-nucleotide amplicon, which was sequenced. The closest match on a blast search in the NCBI GenBank database was rDNA of *Naegleria gruberi*, with only 89 % sequence identity. Normally, a DNA sequence with such a low sequence identity would be dismissed as a contaminant. However, a species of *Naegleria* being the closest match was interesting. This finding prompted a suspicion of primary amoebic meningoencephalitis, taking into account the clinical presentation, travel history, and lack of any other identified microbial agent. Five brain biopsies were secured during the autopsy. DNA extracted from three of these yielded an identical amplicon in the D1/D2 PCR assay as the one found in CSF.

The brain biopsies and CSF were submitted to the Laboratory of Parasitology, Statens Serum Institut (SSI) in Copenhagen for specific PCR assays for free-living amoebae. The SSI, using a previously published multiplex real-time PCR assay targeting *Naegleria fowleri*, *Acanthamoeba* spp. and *Balamuthia mandrillaris* (Qvarnstrom *et al.,* 2006), reported the presence of DNA from *N. fowleri* in all samples, with cycle threshold (Ct) values ranging from 21 to 31. The biopsy yielding the lowest Ct value (and thus the highest DNA copy number) was from the olfactory bulb. Sequencing of amplicons obtained by subsequent conventional PCR using the primers NAEGL-FOR1, NAEGL-SHORT-REV1 and NAEGL-REV1 (Table 1) confirmed the presence of DNA belonging to *N. fowleri*. The patient’s illness was determined to be PAM.

**Table 1.** Primers used for sequencing of *Naegleria fowleri* rDNA

Primer	**Sequence (5′→ 3′)**	Direction	Location
NAEGL-FOR1	AAGGATACCACCGT TAACTGC	Forward	18S rDNA
NAEGL-SHORT-REV1	GGTTATCTACACCCAAATCATGG	Reverse	18S rDNA
NAEGL-REV1	GCCAGGTTCATCTCTTCGT	Reverse	18S rDNA
Nf-Oslo-F1	GCGATTTAGCATGGGACTGC	Forward	18S rDNA
Nf-Oslo-F2	CGTTATGACAGGGATCGAGGATT	Forward	18S rDNA
Nf-Oslo-R2	CCTTGGATTCAGGATGGGCTT	Reverse	28S rDNA
Nf-Oslo-R1	TGTGTAGCCCAATTCCCGTTTA	Reverse	28S rDNA
NL-4*	GGTCCGTGTTTCAAGACG	Reverse	28S rDNA

*Primer NL-4 originally published by Kurtzman & Robnett (1997).

A contiguous 2863-nucleotide sequence reflecting the ribosomal DNA of the *N. fowleri* strain, subsequently assembled by primer walking using additional custom-designed primers (Table 1), has been deposited in the NCBI GenBank database with accession number KT375442.

**Fig. 1.**
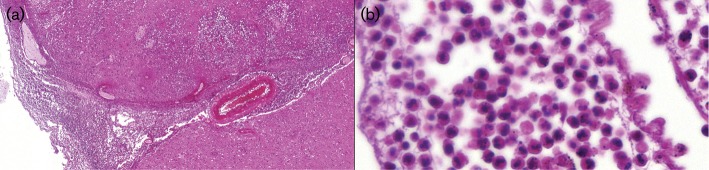
Haematoxylin and eosin (H/E) staining of post-mortem brain tissues showing. (a) acute meningoencephalitis (×5 magnification), and (b) inflammatory infiltrates dominated by polymorphonuclear granulocytes (×60 magnification).

**Fig. 2.**
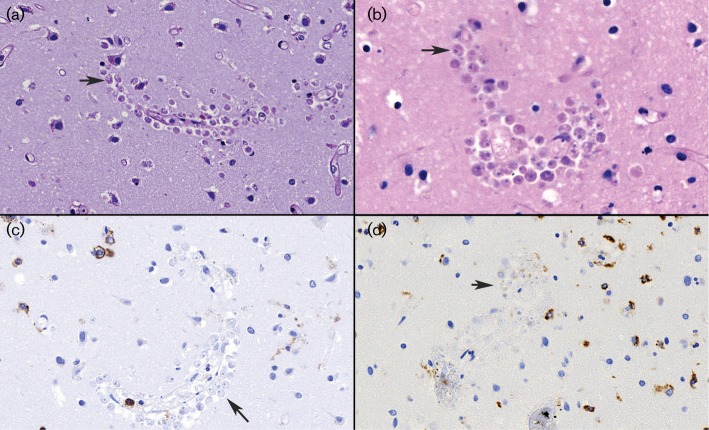
Post-mortem brain tissue sections showing accumulations of rounded structures morphologically consistent with amoebic trophozoites (arrows) around intraparenchymal vessels. (a) PAS stain (×40 magnification); (b) Mucicarmine stain (×60); (c) CD45 immunohistochemistry (×40); (d) CD68 immunohistochemistry (×40).

Autopsy was performed 3 days *post*-*mortem*. Macroscopically, the brain was significantly oedematous, with signs of herniation. The microscopic findings were consistent with acute meningoencephalitis, with acute inflammatory infiltrates dominated by polymorphonuclear granulocytes, in areas with abscess formation in the brain tissue (Fig. 1). Granuloma formation was absent. Accumulations of rounded structures interpreted as morphologically consistent with amoebic trophozoites were visible mostly around intraparenchymal vessels somewhat laterally to the areas with more heavy inflammatory infiltrates (Fig. 2). The structures appeared somewhat smaller than macrophages, contained a clear, halo-like rounded zone with a condensated central point, and were poorly stainable in special stains such as haematoxylin and eosin (H/E), Periodic acid–Schiff (PAS) and Grocotte, as well as immunohistochemically negative for CD45 (pan-leukocyte marker) and CD68 (macrophage marker).

A detailed history was taken from the patient’s husband and sister-in-law, who had participated in the trip to Thailand. They denied bathing in any freshwater, including pools. However, upon direct questioning, they reported that the patient had been carrying out daily nasal irrigation for several years, and had continued doing so while in Thailand, using tap water from the local water supply to which she added a tablespoon of table salt immediately before use. The group rented a privately owned apartment at Jomtien Beach south of Pattaya, connected to a municipal water supply. In addition, they stayed two nights at a hotel on the island of Koh Chang, returning two weeks before the end of the trip (12 days before the onset of symptoms). This hotel is supplied with groundwater from a private well. At both locations, tap water was heated by continuous-flow water heaters.

## Discussion

This is the first imported case of PAM diagnosed in Scandinavia. The patient presented with typical symptoms and signs of PAM, initially mimicking bacterial meningitis, with rapid progression to loss of consciousness, increased intracerebral pressure, herniation and death.

The mechanism of infection is believed to be nasal irrigation using tap water containing *Naegleria fowleri*. The incubation period of PAM is generally short (range 1–9 days) (Capewell *et al.,* 2015; Visvesvara *et al.,* 2007), suggesting that the infection was most likely acquired from tap water in the apartment at Jomtien Beach, as the patient had stayed at this location for the last 12 days before symptom onset. PAM following nasal irrigation with tap water has been reported previously from the USA (Yoder *et al.,* 2012) and Pakistan (Shakoor *et al.,* 2011). A confirmed finding of *N. fowleri* in water from a municipal water supply in Louisiana used for a toy water slide, leading to infection in a child, has also been published (Cope *et al.,* 2015). Although chlorination treatment of drinking water will inhibit *N. fowleri*, the water distribution system in the latter case was found to have dead spots with no detectable residual chlorine, permitting local growth of *N. fowleri*. The presence of similar dead spots in the water supply used by the present patient for nasal irrigation is plausible.

PAM is increasingly recognised as a disease of developing countries (Siddiqui & Khan, 2014). Furthermore, the true incidence of PAM in tropical regions is unknown; the condition is believed to be under-reported due to less available health care and the large number of other, more common, infectious diseases prevalent in these areas (De Jonckheere, 2011; Siddiqui & Khan, 2014). Neither clinicians nor laboratory personnel in Norway have any experience with *Naegleria*, and the initial clinical presentation was typical for bacterial meningitis; as a result, PAM was not initially considered as a differential diagnosis. Even though the travel history was taken into account, as examination for cryptococci is not routinely performed in immunocompetent patients, the event leading to a correct diagnosis was the incidental amplification of DNA from *N. fowleri* by a fungal PCR assay. Interestingly, the examiner reviewing the nigrosin stain of CSF noted the presence of unidentifiable elements that did not resemble cryptococci; these may well have been *N. fowleri* trophozoites.

The key take-home message is that when standard investigations fail to identify a cause of infection in severe meningoencephalitis, it is of crucial importance to continue a broad search for a conclusive diagnosis. PAM should be considered as a differential diagnosis in cases of severe meningitis in patients returning from endemic areas, especially when the clinical presentation and biochemical parameters in CSF clearly suggest meningitis, but no bacteria are detected. *N. fowleri* can be visualised using Giemsa or Wright stains. A wet mount of fresh CSF is also a sensitive method for detection of live trophozoites (Capewell *et al.,* 2015; Siddiqui & Khan, 2014; Visvesvara *et al.,* 2007) and is easily performed in the acute setting with standard equipment, allowing a rapid provisional diagnosis of PAM. Species identification as *N. fowleri* can subsequently be obtained by PCR, or in resource-limited settings, an enflagellation test (Srestha *et al.,* 2015). Rapid diagnosis is paramount: although PAM has a case fatality rate exceeding 95 %, and the prognosis is poor even with treatment, virtually all reported cases of recovery have been in patients who were given early specific therapy administrated intravenously and/or intrathecally (Capewell *et al.,* 2015).

In the absence of an effective therapy, the main focus of a risk management strategy must be prevention. With the knowledge of nasal irrigation as a potential risk factor for PAM, public health advisories should include warnings against the use of freshwater for this purpose in regions where a sufficiently high water quality cannot be assured. As a result of the present case, the Norwegian Institute for Public Health published a warning against the use of tap water for nasal irrigation when travelling abroad. The fatal outcome and the mode of acquisition were subject to substantial coverage in national news media. The case was also reported to Thailand’s International Health Regulation focal point and the European Centre of Disease Control’s Early Warning and Response System.
